# Supplementary material for: Methyl dihydrojasmonate and lilial are the constituents with an "off-label" insect repellence in perfumes
Source: PLoS One. 2018 Jun 19;13(6):e0199386. doi: 10.1371/journal.pone.0199386 (PMC6007898; doi:10.1371/journal.pone.0199386)
Supplement: S1 File — This file contains the original data that generated figures 2–7 and spectral data for methyl dihydrojasmonate. (PDF) [file pone.0199386.s001.pdf]

RAW Data for Figure 2A

Control	DEET	Control	MDJ
22	0	25	2
24	0	29	7
15	0	13	2
15	0	15	2
9	0	7	0
5	0	40	4
38	0	28	13
29	6	21	4
25	1	24	3
22	1	24	2
24	2	31	6
25	3		

Data (%) for Figure 2A

Control	DEET	Control	MDJ
100	0	92.59	7.41
100	0	80.56	19.44
100	0	85.66	13.34
100	0	88.24	11.76
100	0	100	0
100	0	90.91	9.09
100	0	68.29	31.71
82.86	17.14	84	16
96.15	3.85	87.5	12.5
95.65	4.35	92.31	7.69
92.31	7.69	83.78	16.22
89.29	10.71		

RAW Data for Figure 2B

Control	DEET	Control	Lilial
27	0	6	2
21	1	7	3
14	1	7	2
6	1	23	1
19	0	14	3
10	2	21	1
21	2	9	2
18	1	16	3
		13	2
		16	5

Data (%) for Figure 2B

Control	DEET	Control	Lilial
100	0	75	25
95.45	4.54	70	30
93.33	6.66	77.77	22.22
85.71	14.28	95.83	4.166
100	0	82.35	17.64
83.33	16.66	95.45	4.54
91.3	8.69	81.81	18.18
94.73	5.26	84.21	15.78
		86.66	13.33
		76.19	23.8

RAW Data for Figure 2C

Control	DEET	Control	IM
15	0	16	25
22	2	25	20
21	1	25	8
16	0	7	16

Data (%) for Figure 2C

Control	DEET	Control	IM
100	0	39.02	60.97
91.66	8.33	55.55	44.44
95.45	4.54	75.75	24.24
100	0	30.43	69.56

RAW Data for Figure 2D

Control	Galaxolide	Control	Lyral
21	18	13	8
23	11	15	8
25	13	8	7
24	13	15	5
19	15	7	6
14	9	7	4

Data (%) for Figure 2D

Control	Galaxolide	Control	Lyral
53.84	46.15	61.9	38.09
67.64	32.35	65.21	34.78
65.78	34.21	53.33	46.66
64.86	35.13	75	25
55.88	44.11	53.84	46.15
60.86	39.13	63.63	36.36

RAW Data for Figure 3A

Control	DEET	Control	Mixture
26	6	22	8
28	1	17	6
12	1	22	6
8	1	12	3
9	3	10	4
6	2	7	2
31	2	24	11
40	6	31	1
18	6	21	1
25	1	17	1
12	1	11	1
12	0	15	0
		6	0

Data (%) for Figure 3A

Control	DEET	Control	Mixture
81.25	18.75	73.33	26.66
96.55	3.44	73.91	26.08
92.3	7.692	78.57	21.42
88.88	11.11	80	20
75	25	71.42	28.57
75	25	77.77	22.22
93.93	6.06	68.57	31.42
86.95	13.04	96.87	3.12
75	25	95.45	4.54
96.15	3.84	94.44	5.55
92.3	7.69	91.66	8.33
100	0	100	0
		100	0

RAW Data for Figure 3B

Control	DEET	Control	Mixture
10	1	20	4
21	2	16	2
16	3	17	1
18	0	24	2
22	0	20	1
14	0	18	2

Data (%) for Figure 3B

Control	DEET	Control	Mixture
90.9	9.09	83.33	16.66
91.3	8.69	88.88	11.11
84.21	15.78	94.44	5.55
100	0	92.3	7.69
100	0	95.23	4.76
100	0	90	10

Data for Figure 4

DEET	MDJ	Lilial
60	135	13
45	170	10
50	180	8
115	90	14
280	340	19

Data for Figure 5

MDJ	Lilial	MDJ	Lilial
36.17	9.20	50.13	2.03
34.64	9.78	56.42	2.82
36.14	8.66	58.21	2.55

Data for Figure 6

DEET						
T=0	97.37	92.59	100.00	100.00	100.00	100.00
T=2	100.00	100.00	96.97	91.18	94.74	90.00
T=4	98.08	93.75	100.00	90.00	94.44	100.00
T=6	95.83	91.07	95.24	100.00	100.00	100.00

Bombshell						
T=0	89.19	88.89	91.30	93.75	94.12	95.45
T=2	86.11	81.08	91.67	69.23	84.62	93.75
T=4	93.18	93.62	89.66	66.67	78.95	82.35
T=6	70.27	95.24	94.74	94.44	91.43	93.33

Ivanka Trump						
T=0	92.31	96.88	100.00	96.00	100.00	100.00
T=2	92.50	92.86	91.89	88.57	80.65	90.63
T=4	97.78	90.00	94.44	93.10	90.48	89.47
T=6	91.11	97.67	97.56	92.11	97.30	96.88

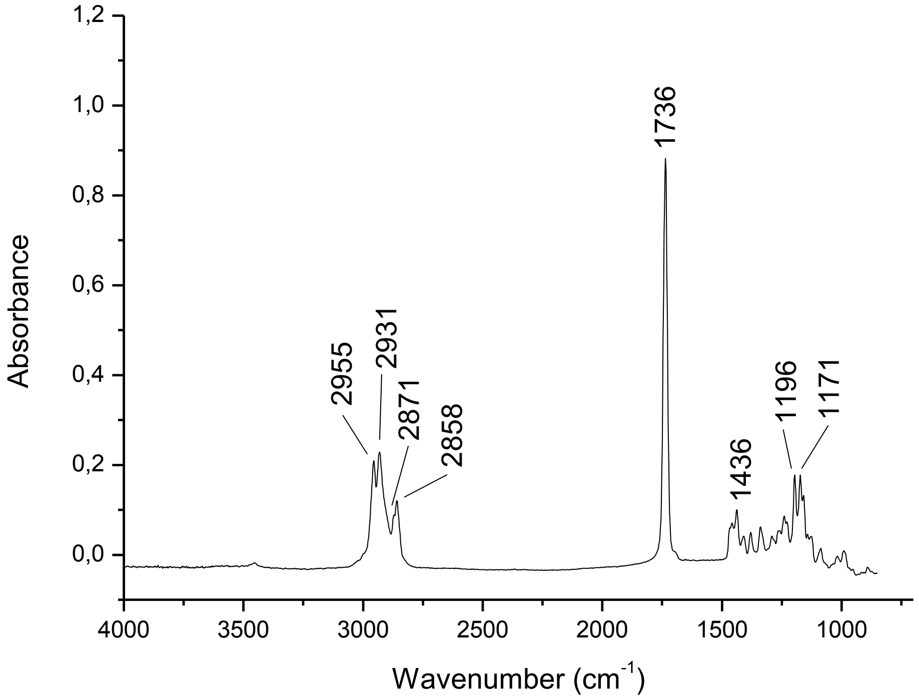

FTIR Data for methyl dihydrojasmonate

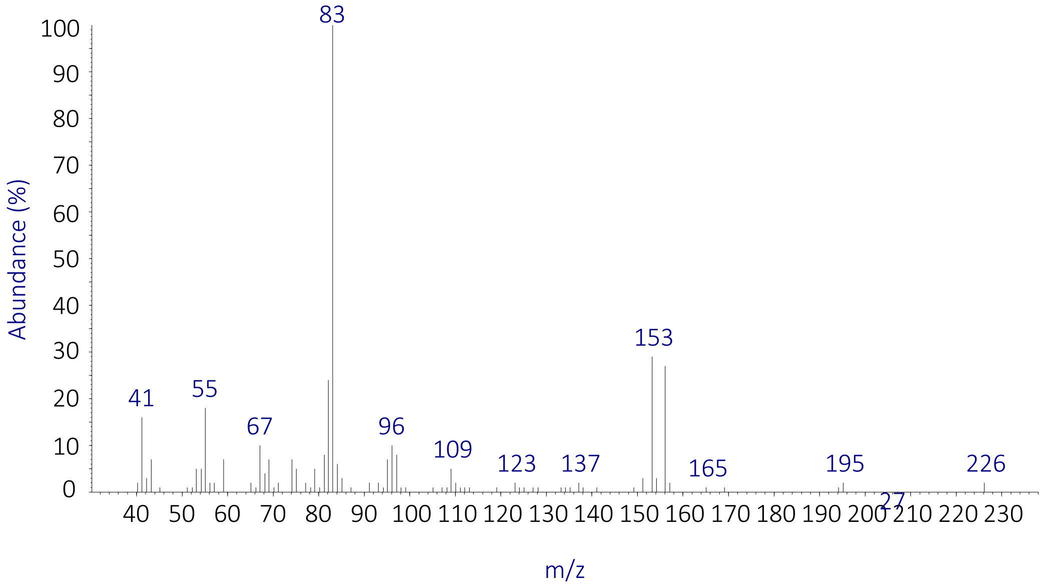

MS data for methyl dihydrojasmonate
